# Prevalence of *Campylobacter* species in milk and milk products, their virulence gene profile and anti-bio gram

**DOI:** 10.14202/vetworld.2015.1-8

**Published:** 2015-01-02

**Authors:** Shivani Modi, M. N. Brahmbhatt, Y. A. Chatur, J. B. Nayak

**Affiliations:** Department of Veterinary Public Health and Epidemiology, College of Veterinary Science & Animal Husbandry, Anand Agricultural University, Anand, Gujarat, India

**Keywords:** antibiotic susceptibility, *Campylobacter jejuni*, polymerase chain reaction, virulence gene

## Abstract

**Aim:**

During the last decades, number of food poisoning cases due to *Campylobacter* occurred, immensely. After poultry, raw milk acts as a second main source of *Campylobacter*. Therefore, the present study was undertaken to detect the prevalence of *Campylobacters* in milk and milk products and to know the antibiotic sensitivity and virulence gene profile of *Campylobacter* spp. in Anand city, Gujarat, India.

**Material and Methods:**

A total of 240 samples (85 buffalo milk, 65 cow milk, 30 cheese, 30 ice-cream and 30 paneer) were collected from the different collection points in Anand city. The samples were processed by microbiological culture method, and presumptive isolates were further confirmed by genus and species-specific polymerase chain reaction using previously reported primer. The isolates were further subjected to antibiotic susceptibility assay and virulence gene detection.

**Result:**

*Campylobacter* species were detected in 7 (2.91%) raw milk samples whereas none of the milk product was positive. All the isolate identified were *Campylobacter jejuni*. Most of the isolates showed resistance against nalidixic acid, ciprofloxacin, and tetracyclin. All the isolates have three virulence genes *cad*F, c*dt*B and *flg*R whereas only one isolate was positive for *iam*A gene and 6 isolates were positive for *fla* gene.

**Conclusion:**

The presence of *Campylobacter* in raw milk indicates that raw milk consumption is hazardous for human being and proper pasteurization of milk and adaptation of hygienic condition will be necessary to protect the consumer from this zoonotic pathogen.

## Introduction

An infection that occurs due to consumption of food of animal origin is an important public health problem in all over the world [1]. Animal products that are mainly used for human consumption are meat of different animal, milk and the products that are made from them. In all these, milk and milk products are mainly used as a dietary source by Indians [2]. Raw milk acts as the main source for various pathogens such as *Escherichia coli*, *Mycobacterium bovis*, *Listeria monocytogenes, Campylobacter, Brucella* and *Salmonella* [3]. In all these, *Campylobacters* the leading cause of zoonotic infections in many countries and the public health burden due to Campylobacteriosis is increasing day to day [4]. It is a gastrointestinal disorder that mainly affects infants, elderly people, patients with underlying disease and immunocompromised individuals. Campylobacteriosis is usually a self-limited disease, and antimicrobial therapy is not generally indicated [5,6].

The family Campylobacteraceae consists of four genera, comprising *Campylobacter*, *Arcobacter*, *Dehalospirilum*, and *Sulfurospirilum*. Under this genus campylobacter consists of 32 species and 13 subspecies [7]. They are motile, curved S- or spiral shaped Gram-negative rods, 0.2-0.8 μm wide and 0.5-5 μm long [7]. In all the species, *Campylobacter jejuni* and *Campylobacter coli* are most important from food safety point of view and causes enteritis in domestic animal and human being [4,6].

*Campylobacters* are inhabitants in the intestinal tract of a wide variety of wild and domestic animals, especially birds [8]. Inadequately cooked meat, particularly poultry, unpasteurized milk, contaminated drinking water, ready to eat food products, direct contact with animals, fecal runoff of domestic animals and birds contaminating surface water act as main source of organism [9-11]. Raw milk is primarily to be contaminated by bovine feces. However, direct contamination of milk as a consequence of mastitis also occurs [12].

It causes diarrhea and abortion in animals. In human being, the gastroenteritis due to *Campylobacter* ranges from mild to severe diarrheal disease. Instead of diarrhea (often bloody diarrhea), other symptoms are cramping, abdominal pain and fever within 2-5 days after exposure to the organism, with symptoms typically lasting 1 week [5]. Complications that occur due to Campylobacter are Guillain-Barre syndrome, reactive arthritis, hemolytic uraemic syndrome and meningitis, etc. [13]. Though infections due to *C. jejuni* are rare, and most patients do not need specific interventions. However, emergence of antimicrobial resistant *Campylobacters* had increased the chances of increased invasive illness [14]. The increased prevalence of this resistant campylobacter has been linked to the illegitimate use of antimicrobials in food animals, animal feeds and flock treatment of animals rather than the individual approach [15].

Despite the increased recovery of *Campylobacters* as a food borne pathogen, the specific virulence and pathogenic mechanisms by which microaerophilic *Campylobacter* species causes infection are still poorly understood [16]. The putative virulence factor for adhesion and invasion of epithelial cells, toxin production, and flagellar motility are thought to be important virulence mechanisms[17]. But, different studies have indicated that different virulence marker could play a role of colonization, adherence, and invasion of *Campylobacter* spp. in the animal and human being.

*Campylobacter* infection are sporadic in nature and have worldwide occurrence [18-23]. In UK, it is the principal cause of gastroenteritis while in United States; it is fifth domestically acquired foodborne infection [24]. It is the most common notifiable foodborne disease in Austria, Denmark, Finland, Germany, Italy, Sweden, and Norway [25,26]. Due to less information about *Campylobacter* in milk and milk products in India, this study was projected to characterize the *Campylobacter* isolates from milk and milk products to know the prevalence and antibiotic resistance pattern of *Campylobacter* spp. in Anand city, Gujarat, India.

## Material and Methods

### Ethical approval

The study entailed the collection of milk samples from farmer’s cattle milk from collection points, retail shop and vendor. Ethical approval was obtained Ethical Review Committee, Veterinary Science College, AAU, Anand, Gujarat. Farmers, person in charge of units and shops were informed about study and verbal consent was taken before collection of samples.

### Source of experimental samples

A total of 240 samples comprising raw milk (85 buffalo and 75 cow milk), cheese (30), ice-cream (30) and paneer (30) were collected from different collection points, retail shops and vendors in and around the Anand city in sterilized container in ice pack and processed within 2 h.

### Enrichment and plating of milk and milk product samples

Samples were processed to isolate the *Campylobacter* spp. as per the method described by Salihu *et al*. [27]. In brief, pH of milk samples was adjusted at 7.5 and 20 ml of milk was centrifuged at 14,000 rpm for 20 min. at 4°C. The pellet was suspended in 45 ml of Preston enrichment broth base containing Preston enrichment supplement, *Campylobacter* growth supplement (HiMedia Laboratories, Mumbai, India) and 5% (v/v) defibrinated sheep blood in 100 ml sterile screw cap flask. For dairy product samples, 25 g of samples were homogenized in normal saline and transferred to 225 ml of Preston enrichment broth base containing *Campylobacter* selective supplement IV (HiMedia Laboratories, Mumbai, India) and 5% (v/v) defibrinated sheep blood, mixed properly and incubated at microaerophilic environment (85% N_2_, 5%O_2_ and 10% CO_2_) in water jacketed CO_2_ incubator (NUAIRE, Polymouth, MN, USA) at 42°C for 42 h. After enrichment culture, a loopful of enriched culture was streaked on blood free charcoal cefoperazone deoxycholate agar medium plates [27]. For selective isolation typical due drop like colonies were isolated on blood agar with 5% defibrinated sheep blood to obtain pure cultures. The inoculated plates were incubated at microaerophilic environment (85% N_2_, 5%O_2_ and 10% CO_2_) at 42°C for 48 h.

### Presumptive identification of isolates

Three or four *Campylobacter*-like colonies were picked from each plate and subjected to gram staining and oxidase, catalase, indoxyl acetate, hippurate hydrolysis test, H_2_S production and nitrate reduction test [21,28].

### DNA extraction

The DNA was extracted by heat and snap chilling method. The two to three colonies of fresh bacterial growth on culture medium was collected, suspended in nuclease-free demonized water and heated at 95°C for 10 min. The samples were cooled immediately and centrifuge for 5 min at room temperature. The supernatant was separated, and 3 µl was used as DNA template.

### Confirmation and species identification of isolates using polymerase chain reaction (PCR)

The biochemically identified isolates were further employed for confirmation as genus *Campylobacter* and species *C. jejuni* and *C. coli*, by polymerase chain reaction amplifying specific target gene using genus and species-specific oligonucleotide primers. The primer sequence and size of target PCR product is shown in Table-1. The DNA amplification for each primer pair was carried out in a Applied Biosystems 2720 Thermal Cycler in 25 µl reaction containing 3 µl of DNA template, 12.5 µl mastermix (Thermo Scientific, USA) (containing 0.05 unit/µl Taq DNA Polymerase, reaction buffer, 4 mM MgCl_2_, 0.4 mM of each dNTP), 10 pmole of each forward and reverse primer (10 pmole/µl), and 7.5 ml nuclease free water with positive and negative control. The control strains of *C. jejuni* and *C. coli* isolates in our department were used for positive control whereas DNase free distilled water was used for negative control. The cycling protocol for the genus confirmation was standardized to set the PCR assay as initial denaturation at 95°C for 5 min followed by 35 cycles with denaturation at 95°C for 30 s, annealing at 53°C for 30 s and extension at 72°C for 30 s and final extension at 72°C for 10 min. For hippuricase (*hip*O) gene of *Campylobacter jejuni*, cycling condition was optimized to initial denaturation at 95°C for 5 min, followed by 35 cycles with denaturation at 95°C for 45 s, annealing at 51°C for 45 s and extension at 72°C for 45 s and final extension at 72°C for 7 min and for asperkinase A (*ask*A) gene of *C. coli* cycling condition was optimized to initial denaturation at 95°C for 5 min followed by 35 cycles with denaturation at 95°C for 30 s, annealing at 53°C for 30 s and extension at 72°C for 30 s and final extension at 72°C for 7 min. On completion of the reaction, the amplified products were held briefly at 4°C. Amplification of the PCR products were detected by electrophoresis in 1.5% agarose gel with ethidium bromide (10 µg/ml) in 0.5X TBE buffer (Sigma, USA) at 100 V for 40 min and documented in G: BOXF3 (SynGene, USA).

**Table 1:** List of the genus and species specific primers.

Gene name	Primer Sequence	Target gene	Amplicon size (bp)	Reference
C412F	GGATGACACTTTTCGGAGC	*Campylobacter* genus 16S rRNA	816	78
C1228R	CATTGTAGCACGTGTGTC			
Hip O1	AGCTAGCTTCGCATAATAACTTG	*C. jejuni* Hippurase Gene	735	79
Hip O2	GAAGAGGGTTTGGGTGGT			
CC1	GGTATGATTTCTACAAAGCGAG	*C. coli* *Asperkinase* gene	500	79
CC2	ATAAAAAGACTATCGTCGCGT			

C. coli=Campylobacter coli, C. jejuni=Campylobacter jejuni

### In vitro antimicrobial drug resistance pattern

All the *Campylobacter* isolates were subjected for antibiotic susceptibility test by Kirby-Bauer disc diffusion method according to the Clinical Laboratory Standards Institute (CLSI) [29] for seven antibiotics as ciprofloxacin (5 mcg), chloramphenicol (30 mcg), nalidixic acid (30 mcg), erythromycin (15 mcg), gentamicin (10 mcg), Streptomycin sulphate (30 mcg) and Tetracycline (30 mcg) as suggested by External Quality Assurance System [30]. Isolates were grown on nutrient broth No. 2 at 37°C for 24 h. The individual broth culture was then smeared on the surface of Mueller-Hinton agar (Hi Media) supplemented with 5% defibrinated sheep blood with the help of sterile cotton swab. The plates were allowed to dry for few minutes. Antibiotic disc was placed on the agar surface within 15 min of inoculation of the plates. The plates were incubated overnight at 37°C. Sensitivity or resistance of an isolate for a particular antibiotic was determined by measuring the diameter of the zone of growth inhibition. The result was interpreted as sensitive, intermediate or resistant by comparing with manufacturer’s instructions. Culture of ATCC 33560 was used to check the quality for antibiotic sensitivity test.

### Virulence gene characterization of Campylobacter isolates

The confirmed isolates of *Campylobacter* species were characterized for *in vitro* detection of virulence genes by PCR for five well-known virulence gene encoding flagellin gene (*fla*A) [31], *campylobacter* adherence gene (*cad*F) [32,33], invasion associated marker, *iam*A [34], flagellar synthesis and modification, *flg*R [16] and cytolethal distending toxin subunit B gene (*cdt*B) [35]. The details of primers for target virulence genes and PCR conditions are described in Table-2 and 3, respectively. The DNA of virulence gene positive control strains available in our department was used in PCR for detection of virulence genes while for negative control DNA template was replaced with nuclease-free distilled water. The positive control PCR revealed PCR product of appropriate size and in a negative control, no product was amplified.

**Table 2:** Oligonucleotide sequence of virulence genes.

Target gene	Primer	Primer Sequence (5’→3’)	Amplicon size (bp)	Reference
Flagellin gene	fla 1	GGATTTCGTATTAACACAAATGGTGC	1725	80
	fla 2	CTGTAGTAATCTTAAAACATTTTG		
*Campylobacter* adherence gene	cad F	TTGAAGGTAATTTAGATATG	400	66
	cad R	CTAATACCTAAAGTTGAAAC		
Invasion associated marker	iam F	GCGCAAAATATTATCACCC	518	34
	iam R	TTCACGACTACTATGCGG		
Flagellar synthesis and modification, *flg*R	JL 1225	GAGCGTTTAGAATGGGTGTG	390	16
	JL 1226	GCCAGGAATTGATGGCATAG		
Cytolethal distending toxin SubunitB gene	CdtB-F	GTTGGCACTTGGAATTTGCAAGGC	495	74
	CdtB-R	GTTAAAATCCCCTGCTATCAACCA		

**Table 3:** PCR cyclic condition of virulence genes.

Gene	Initial denaturation	35 cycles			Final extension

		Denaturation	Annealing	Extension	
Flagellin gene	94°C	94°C	52°C	72°C	72°C
	5 min	45 s	45 s	1 min	10 min
*Campylobacter* adherence gene	94°C	94°C	45°C	72°C	72°C
	5 min	1 min	45 s	1 min	8 min
Invasion associated marker	94°C	94°C	52°C	72°C	72°C
	5 min	1 min	1 min	1 min	5 min
Flagellar synthesis and modification, *flg*R	95°C	95°C	54°C	72°C	72°C
	5 min	1 min	1 min	1 min	5 min
Cytolethal distending toxin subunit B gene	95°C	95°C	57°C	72°C	72°C
	5 min	30 s	30 s	30 s	10 min

PCR=Polymerase chain reaction

## Results and Discussion

Cultural plates that show typical dew drop like colonies were identified as *Campylobacter*. All the isolates were Gram-negative, spiral, curved or S-shaped rods, motile with characteristic darting screw type motility and showed oxidase and catalase positive reactions. All the isolates were positive for hippurate hydrolysis, indoxyl acetate, H_2_S production, nitrate reduction and growth were further confirmed by genus-specific PCR by generating 816 bp of amplicon 16S rRNA sequence and species specific PCR by targeting 735 bp and 500 bp amplicon of hippurase (*hip*O) and asperkinase (*ask*A) gene for *C. jejuni* and *C. coli*, respectively.

The overall 2.91% prevalence of Campylobacter was observed in total of 240 samples processed comprising 150 raw milk, 30 cheese, 30 paneer and 30 ice-cream. All the positive samples were obtained from raw milk (4.66%), none of the milk product sample was found positive for Campylobacter. All the seven *Campylobacter* isolates were identified as *C. jejuni* (100%) by species-specific PCR indicating that this species is distributed widely in the study area. The findings of the present study is concurrent with reports of Kazemeini *et al*. [36], Wysok *et al*. [37] and Rahimi *et al*.[38] where they observed almost similar prevalence rate i.e. 2.5%, 4.6% and 2.32%, respectively. A lower prevalence rate of 1.5% and 1.41% was observed by Lovett *et al*. [39] in Cincinnati, Ohio, Canada and Elango *et al*. [40] in Chennai, India whereas some authors have not detected campylobacters in milk samples [41,42]. On the other hand, higher prevalence rate of 34%, 12.3%, 10.2%, 61%, 12.5%, 12.5%, 66.8%, 3.06% and 8.07% was observed by Wicker *et al*. [43], Jayarao *et al*. [44], Hussain *et al*. [10], Martin *et al*. [45], Khanzadi *et al*. [46], Giacometti *et al*. [47], Mabotel *et al*. [48], Serraino *et al*. [49] and Giacometti *et al*. [50], respectively.

In the case of milk products, we did not found any positive sample which was concurrent with the study of Singh *et al*. [42] who could not detect campylobacters in cheese. In contrast to this Giacometti *et al*. [50] and Jain and Shrivastava [51] found the prevalence rate of 5.0% and 18.33% from traditional cheese while Vaishnavi *et al*. [52] identified the prevalence of 17.2% from paneer.

Although there is significant variation of the presence of *Campylobacter* in different food products as reported by different workers, milk act as a second main source of *Campylobacter*. The present study revealed that *Campylobacter* could be mainly transmitted through milk in comparison to milk products. The possible reason behind this may be destruction of an organism during the processing of milk products. It is mainly destroyed during the boiling of milk, so there are less chances of contamination of this pathogen in milk products. Contamination of milk products is only possible because of unhygienic conditions during the preparation of milk products.

In the present study, all the Campylobacter isolates were resistant to Nalidixic acid (100%), whereas 6 (85.71%) and 1 (14.29%) isolates were resistant to ciprofloxacin and tetracyclin, respectively. One isolate was intermediate while 5 (71.42%) isolates were sensitive to Tetracycline. All the isolates were sensitive for chloramphenicol, gentamicin, streptomycin sulfate and erythromycin. Only one isolates was sensitive for ciprofloxacin. The result is also in collaboration with Chatur *et al*. [53] who observed extremely high resistance of *C*. *jejuni* isolates to nalidixic acid and ciprofloxacin in study area. The presence of quinolone resistant campylobacter strains in the study area indicates the mutation in the gyrase subunit A gene that could be caused due to the treatment of animal with quinolones or their use in animal feed. As environment and water could act as the main source of contamination of milk due to unhygienic conditions, the presence of *Campylobacter* spp. from other animal species cannot be overruled. Looking into the increasing importance of *Campylobacter* sporadic outbreaks, this study suggests regular surveillance program for the detection and understanding the behavior of campylobacters. In Poland, Wysok *et al*. [37] and in Iran, Rahimi *et al*. [38] also obtained the same resistance pattern in their study while Murphy *et al*. [54] and Bopp *et al*. [55] found higher resistance against tetracycline.

**Figure-1:**
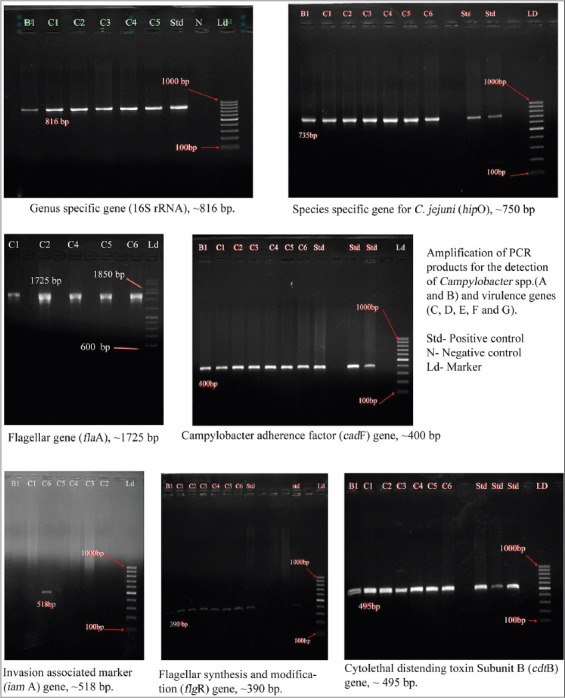
Amplification of PCR products for the detection of *Campylobacter* spp. and virulence genes

*In vitro* detection of virulence genes revealed presence of *Cad*, *Cdt*B and *flg*R genes in all the isolates while only one isolate was positive for *iam*A gene and 6 isolates were positive for *fla*A gene (Figure-1). The Flagellin gene encodes for a flagella protein that is responsible for motility, colonization of gastrointestinal tract and invasion of host cells [31]. Wegmuller *et al*. [56] detected only 6 (6.5%) positive samples out of 93 samples in their study which was much lower in comparison to present study. This could be because of different primers used in this and present study. *Campylobacter* species are motile by means of a single polar, unsheathed flagellum at one or both ends of the organism. Two genes, *fla*A and *fla*B, that are involved in the expression of the flagellar filament have been identified in *C. jejuni* [57,58] and *C. coli* [59]. In both species, the two genes are arranged head-to-tail in the same direction, separated by 174 bp having separate promoter region. In strains like *C. jejuni*, 81116, only *fla*A is expressed [58], whereas in *C. coli* VC167, some *fla*B is also expressed [59]. The *fla*A mutant strains of *C. jejuni* has shown reduced motility and colonization [60,61]. The PCR can detect either one *fla* gene or two, depending on which primers are used. The primers are designed to bind strongly to conserved sequences, but the sequence in between the primers is highly variable and the primers were found to work for *C. coli* and *C. upsaliensis* as well [62]. Though, primer strongly binds in conserved sequences, we could not amplify the approximate 1725 bp PCR product of *fla*A gene using the cited primers in one isolate (C3) obtained from cow raw milk. The mutation could have occurred in primer binding site on *fla*A gene may be due to sub culturing, or already mutant *C. jejuni* isolate in milk sample.

Presence of campylobacter adherence factor in all the isolates was in conformity with the report of Elango *et al*. [40] while it was higher as compared to the finding of Khanzadi *et al*. [46]. They observed the 15.5% occurrence of *cad*F gene among all *Campylobacter* isolates and only 8% in *C. jejuni* isolates. The results of present study agree with the data obtained by other authors, who examined and found 100% prevalence of *Cad* gene in *Campylobacter* isolates derived from different sources [63-66].

Among 7 isolates studied only one (14.28%) isolates were positive for the presence of *iam*A gene and yielded the DNA fragment of 518bp. There was no any previous study for the presence of *iam*A gene in *Campylobacter* isolates that were isolated from milk but this gene was reported by different authors in those *Campylobacter* isolates that were obtained from meat or other sources [67-70].

All the seven isolates were positive for the presence of *flg*R gene and yielded the DNA fragment of 390bp. No any study was conducted previously for the presence of *flg*R gene in *Campylobacter* isolates isolated from milk. This gene encodes the signal-transduction regulatory protein responsible for flagellar synthesis and modification as a gene in response regulator of a two component system (*Flg*R/S) and described by different authors responsible for phase variation is a mechanism whereby the bacteria can modify the antigenic make-up of its surface to evade the host immune system or adapt to new hosts or environments [71-73].

All the seven isolates were positive for the presence of *cdt*B gene and yielded the DNA fragment of 495bp. The absence of reports of screening of the *Campylobacter* isolates isolated from milk and milk products for the presence of *cdt*B gene was the limitation for us to compare the result of the occurrence of *cdt*B gene in this study. On the contrary, most of authors have detected this gene in Campylobacter isolates isolated from other food and clinical samples [74,75] whereas it has also been detected in cattle beef and fecal samples [74,76,77].

## Conclusions

Considering the zoonotic potential of this organism, in this study, we conclude that the raw milk and milk products act as the main source of *Campylobacter*, which is the leading cause of gastroenteritis worldwide. Due to its thermophilic nature, potential pathogenic character and the capability to grow in milk, monitoring and surveillance of this pathogen in raw milk and milk product should be necessary. It has been necessary to increase the activities for development of the concept for production of safe milk; maintain the hygienic condition at farm and processing unit and pasteurization of milk to protect the consumer.

## Author’s Contributions

SM supervised the overall research work. YAC and SM participated in sampling, analysis of samples and made available relevant literatures. MNB and JBN participated in draft and revision of the manuscript. All authors read and approved the final manuscript.
